# The role of Thyroid Transcription Factor-1 and Tumor differentiation in Resected Lung Adenocarcinoma

**DOI:** 10.1038/s41598-017-14651-y

**Published:** 2017-10-27

**Authors:** Tsai-Wang Huang, Ke- Feng Lin, Chien-Hsing Lee, Hung Chang, Shih-Chun Lee, Yi-Shing Shieh

**Affiliations:** 1 Graduate Institute of Medical Science, National Defense Medical Center, Taipei, Republic of China; 2 Division of Thoracic Surgery, Division of Thoracic Surgery, Taipei, Republic of China; 3 Graduate Institute of Applied Science and Technology, National Taiwan University of Science and Technology, Taipei, Republic of China; 4 Department of Internal Medicine, Tri-Service General Hospital, National Defense Medical Center, Taipei, Republic of China; 5 Department of Dentistry, Tri-Service General Hospital, National Defense Medical Center, Taipei, Republic of China

## Abstract

To investigate the role of thyroid transcription factor-1 (TTF-1) and tumor differentiation in resected lung adenocarcinoma. A total of 520 patients with clinical early stage lung adenocarcinoma who underwent surgical resection were reviewed retrospectively. Clinical data and outcomes were evaluated with an average follow-up of 117 months. The results were validated via lung cancer cell line studies. The clinical parameters did not differ between relapse and nonrelapse patients. Exceptions were tumor differentiation, lymphovascular space invasion, F^18^-fluorodeoxyglucose maximum standard uptake value, tumor size, and pathological stage (*p* < 0.001). Poor tumor differentiation was the independent prognostic factor (odds ratio: 2.937, *p* = 0.026). The expression of TTF-1 was correlated with tumor differentiation in resected lung adenocarcinoma patients (*p* < 0.001). Five-year survival was 60.0% for score 1 TTF-1 expression patients, 80.1% for score 2 TTF-1 expression patients, and 86.1% for score 3 TTF-1 expression group patients. The lung cancer cell line study of knockdown and overexpression of TTF-1 revealed TTF-1 mediated High Mobility Group AT-Hook 2 (HMGA2) protein involved with epithelium-mesenchymal transformation. The chromatin immunoprecipitation revealed TTF-1 regulated HMGA2 via direct binding. TTF-1/HMGA2 axis was associated with tumor differentiation and mediated the aggressiveness of the tumor and prognosis.

## Introduction

Lung cancer remains the leading cause of cancer-related death. Despite advances in therapeutic drugs and newly developed target therapies, long-term survival is still unsatisfactory. Mortality following surgical resection is thought to be associated with tumor relapse^1,2^. Many studies have investigated the clinical and basic parameters associated with relapse after surgical resection of lung cancer. Tumors graded as poorly differentiated are found to be an independent prognostic factor in patients with stage I non-small-cell lung cancer (NSCLC)^3,4^, but the mechanism is not clear.

Thyroid transcription factor-1 (TTF-1), a member of the homeodomain-containing transcription factor family, activates the expression of selected genes in the thyroid, lung, and brain^5^. TTF-1 is known to play key roles in the control of embryonic development and differentiation^6,7^. The expression of TTF-1 is restricted to alveolar type II cells^8^. It has been found in all types of lung cancer but is reported frequently in adenocarcinoma^9,10^. TTF-1 has been shown to be a favor prognostic factor for survival in NSCLC^11^. Some studies have shown that differentiation grade is not a prognostic factor for lung cancer survival^12,13^. A dual role of TTF-1 expression in lung adenocarcinoma has been reported^14^. The mechanisms which control tissue-specific expression of TTF-1, tumor differentiation, and their prognostic significance are not fully understood. One study showed the possible mechanism of suppression of lung adenocarcinoma progression by TTF-1 via regulation of High Mobility Group AT-Hook 2 (HMGA2) protein^15^. We, therefore, aimed to clarify the role of TTF-1 in tumor differentiation and the aggressiveness of lung adenocarcinoma.

## Materials and Methods

### Patients and Protocol

This study was approved by the Institutional Review Board of our hospital (TSGHIRB 1-105-05-010), and patient consent was waived. All the methods were performed in accordance with relevant guideline. We retrospectively evaluated the records of patients who underwent surgical resection for clinical early stage (I&II) lung adenocarcinoma from January 2002 to June 2010 in Tri-Service General Hospital, Taiwan. The cancer staging work-up included chest & upper abdomen computed tomography (CT) and positron emission tomography (PET) scans. Mediastinal staging procedure including mediastinoscopy or Endobronchial ultrasound with transbronchial needle biopsy(EBUS-TBNA) was done if suspicious mediastinal lymphadenoapthy (short-axis diameter >1.0 cm was identified on CT scan or light up on PET scan). All patients underwent surgical resection and systemic lymph node dissection. Patients were excluded if they had synchronous cancer, second primary lung cancer, preoperative chemotherapy, or died within 30 days of operation. Selected patients were staged according to the seventh edition of the American Joint Committee on Cancer adopted in 2009^16^. A total of 520 patients were enrolled. Postoperative surveillance included contrast-enhanced chest CT, serum carcinoembryonic antigen (CEA) levels with 4 months interval. The magnetic resonance imaging of the brain was performed when neurological symptoms developed. Relapse was documented with either imaging or pathological diagnosis. The immunohistochemical stain (IHC) of TTF-1 for each specimen was reviewed by experienced pathologists with grading. The degree of TTF-1 IHC staining were scored by following criteria: Score 1: less than 1%; Score 2: 1–49%; Score 3: 50–100%^17^.

### Cell Lines and Cell Culture

Human lung cancer cell lines, CL1-0, CL1-5, A549, and H1299 (gift in kind from Pro. Cheng-Wen Wu of the Institute of Biomedical Sciences, Academia Sinica, Taiwan) were maintained in RPMI-1640 (Gibco, Grand Island, NY, USA) supplemented with 10% fetal bovine serum and 1% penicillin–streptomycin–amphotericin (Biological Industries, Kibbutz Beit-Haemek, Israel). Cells were cultured in a standard humidified incubator at 37 °C in a 5% carbon dioxide atmosphere, and the medium was changed twice each week. Cells were grown until they were 80–90% confluent. All cultures were negative for *Mycoplasma* infection.

### RNA Extraction and Reverse Transcription-Polymerase Chain Reaction

Total RNA was isolated from cultured cells using TRIzol reagent (Thermo Scientific Life Sciences, Carlsbad, California, USA) following the manufacturer’s protocol. cDNA synthesis was performed using a cDNA reverse transcription kit (ABI, Foster City, CA, USA). In the reverse transcription–polymerase chain reaction (RT–PCR) step, 50 pmol of each forward and reverse primer was used. Standard RT–PCR amplification conditions were applied as follows: 95 °C for 5 min, followed by 30 cycles of 95 °C for 30 s, 58 °C for 30 s, and 72 °C for 30 s, and a final amplification at 72 °C for 5 min. Each measurement was performed in duplicate, and the threshold cycle value was determined for each amplification curve. The geometric mean of endogenously expressed glyceraldehyde-3-phosphate dehydrogenase was used to normalize the expression. RT–PCR products were separated on 2% agarose gels and visualized with Health View nucleic acid stain under ultraviolet light.

### Protein Extraction and Western Blotting

Tissues and cells were washed with ice-cold phosphate-buffered saline and lysed with cell lysis buffer containing protease inhibitors (50 mM Tris [pH 7.6], 150 mM NaCl, 1% NP-40, and 0.1% sodium dodecyl sulfate [SDS]). Cell lysates were clarified by centrifugation at 14,000 *g*, 4 °C for 15 min. The protein concentration of cell lysates was measured using a bicinchoninic acid assay (Thermo Scientific, Rockford, IL, USA). Western blotting was performed using an SDS-polyacrylamide gel electrophoresis system. Briefly, protein samples were resuspended in sample buffer containing 10% β-mercaptoethanol and electrophoresed on a 10% polyacrylamide gel. Proteins were transferred electrophoretically to a polyvinylidene fluoride membrane, and nonspecific binding sites were blocked by immersion of the membrane in 5% skim milk. The membrane was incubated with primary antibodies against TTF-1 (1:500, SC-53136, Santa Cruz, Dallas, USA), HMGA2 (1:500, #5269, Cell Signaling, Danvers, MA, USA), EGFR (1:1000, #2116 S, Epitomics, Burlingame, USA), E-cadherin (1:1,000, #610182, BD, San Jose, CA,USA), ZO-1 (1:1,000, #610966, BD, San Jose, CA,USA), Vimentin (1:500, #5741, Cell Signaling, Danvers, MA, USA), Fibronectin (1:500, ab32419, Abcam, Cambridge, UK), phosphor-EGFR (1:25; ab134005, Abcam, Cambridge, UK). GAPDH (1:5000, #5147, Cell Signaling, Danvers, MA, USA) was used as the loading control. The secondary antibodies were horseradish peroxidase-conjugated goat anti-mouse and goat anti-rabbit antibodies (Jackson Immuno Research, West Baltimore Pike, USA). Protein was detected with Western blotting Enhanced chemiluminescence reagents (Advansta, Kaysville, Utah, USA) using an ultraviolet lamp (Thermo, Wilmington, DE, USA).

### RNA interference

Short hairpin RNA (shRNA) was designed and purchased from National RNAi Core Facility, Institute of Molecular Biology/Genomic Research Center, Academia Sinica. The sequence of shRNA used for targeting TTF-1 was 5′-ACACTGAGAACGGATTTTT-3′. The RNAi interference processes were performed by Polyjet transfection reagents (SignaGen Laboratories, Gaithersburg, MD, USA). Briefly, cells were seeded at a density of 2 × 10^5^ in 6-well culture plates and incubated overnight. The 1 μg of shRNA was transiently transfected into cells for 24 hr and followed by changing with the complete medium. On the third day, the cells were used for further experimental analysis.

### Ectopic expression of TTF-1

The human TTF-1 cDNA ORF clone was designed and obtained from Origene Technologies, Rockville, MD, USA. Briefly, cells were seeded at 2 × 10^5^ in 6-well culture plates and incubated overnight. The human TTF-1 cDNA ORF clone was transiently transfected into cells for 24 hr using Polyjet transfection reagents (SignaGen Laboratories, Gaithersburg, MD, USA) and followed by changing with the complete medium. Following 48 hr incubation, the cells were utilized for following experimental analysis.

### Transwell invasion assay

Invasion ability was investigated by using Transwell invasion chambers. The culture inserts (8-μm pore size; BD Biosciences, Franklin lakes, NJ, USA) was precoated with 30 μg/insert Matrigel (BD Biosciences, Franklin lakes, NJ, USA) on top of the membrane. A total of 2.5 × 10^4^ cells were seeded onto the upper chamber in medium containing 10% NuSerum. The lower chamber contained complete medium (RPMI supplemented with 10% FBS). The cells were allowed to invade toward the lower chamber overnight. On the second day, the invasive cells were fixed with methanol, stained with 50 μg/mL propidium iodide (Sigma, St. Louis, MO, USA) in ddH_2_O and analyzed using immunofluorescent microscopy. The amounts of invasive cells were quantified with ImageJ software.

### Quantification of miRNA expression

The MicroRNAs was extracted, purified, and converted to cDNA using TaqMan^TM^ Advanced miRNA cDNA Synthesis Kit (Thermo Fisher Scientific, Waltham, MA, USA). The expression of miR-33a was quantified by quantitative real time polymerase chain reaction (qPCR). The miR-33a primer was designed and synthesized by Origene Technologies, Rockville, MD, USA. qPCR was performed in a 20 μl reaction solution containing of 4 μl cDNA, 4 μl ddH_2_O, 1 μl of each primer, and 10 μl of SYBR Green I master mix (Roche, Basel, Switzerland) using the Roche LightCycler 480 Real-Time PCR system (Roche Applied Science, Penzberg, Bavaria, Germany).

### Chromatin Immunoprecipitation

The ChIP assay was performed according to the instructions of Pierce Magnetic ChIP kit (Pierce, Rockford, IL, USA). Briefly, cells were cross-linked by the addition of 16% formaldehyde to obtain a final concentration of 1% formaldehyde for 10 minutes at room temperature, and the reaction was terminated by the incubation with 1X glycine for 5 minutes at room temperature. Subsequently, the crosslinked cells were lysed with membrane extraction buffer containing proteinase/phosphatase inhibitor for 10 minutes on ice. Genomic DNA was fragmented into 200 bp segments using the combination of Micrococcal Nuclease and Bioruptor USD-200TM sonicator (Diagenode, Liege, Belgium). 10% of digested chromatin was used for input control and the remaining sample was further incubated with 5 μg TTF-1 antibody or normal rabbit IgG with rotation at 4 °C overnight, respectively. After incubation, 20 μL of the magnetic beads were added into each sample and incubated for 2 hours at 4 °C with mixing. The magnetic beads were washed sequentially with provided wash buffer and pelleted by centrifuging at 3000 × g for 5 minutes. The chromatin was eluted from the magnetic beads by incubation with elution buffer at 65 °C for 30 minutes with vigorous shaking. DNA-protein cross-links reversal was performed by using a high salt solution containing 6 μL of 5 M NaCl and 2 μL of 20 mg/mL Proteinase K. The precipitated DNA was recovered using the DNA Clean-Up Column and collection tube and eluted with 30 μL of DNA Column Elution Solution. PCR was performed using the primers designed to recognize HMGA2 promoter containing TTF-1 recognition site, which were5′-CCCTTGTTATAAATTCAAAAGGGG-3′and 5′-GGTAACGTGTACAGAGGTCAGCAA-3′.

### Statistical Analysis

Descriptive data are expressed as mean ± standard deviation. Student’s *t* test was used to investigate continuous variables, and the χ^2^ test was used to compare categorical variables between groups. Overall survival (OS) and disease-free survival (DFS) were calculated using Kaplan–Meier survival analysis. Multiple logistic regression analysis was used to identify independent risk factors for patients with relapse. For statistical analysis, each cell experiment was performed in triplicate and repeated at least three times. SPSS 14.0 software (SPSS, Inc., Chicago, IL, USA) was used for all analyses, and statistical significance was defined as *p* < 0.05.

## Results

**Figure 1:**
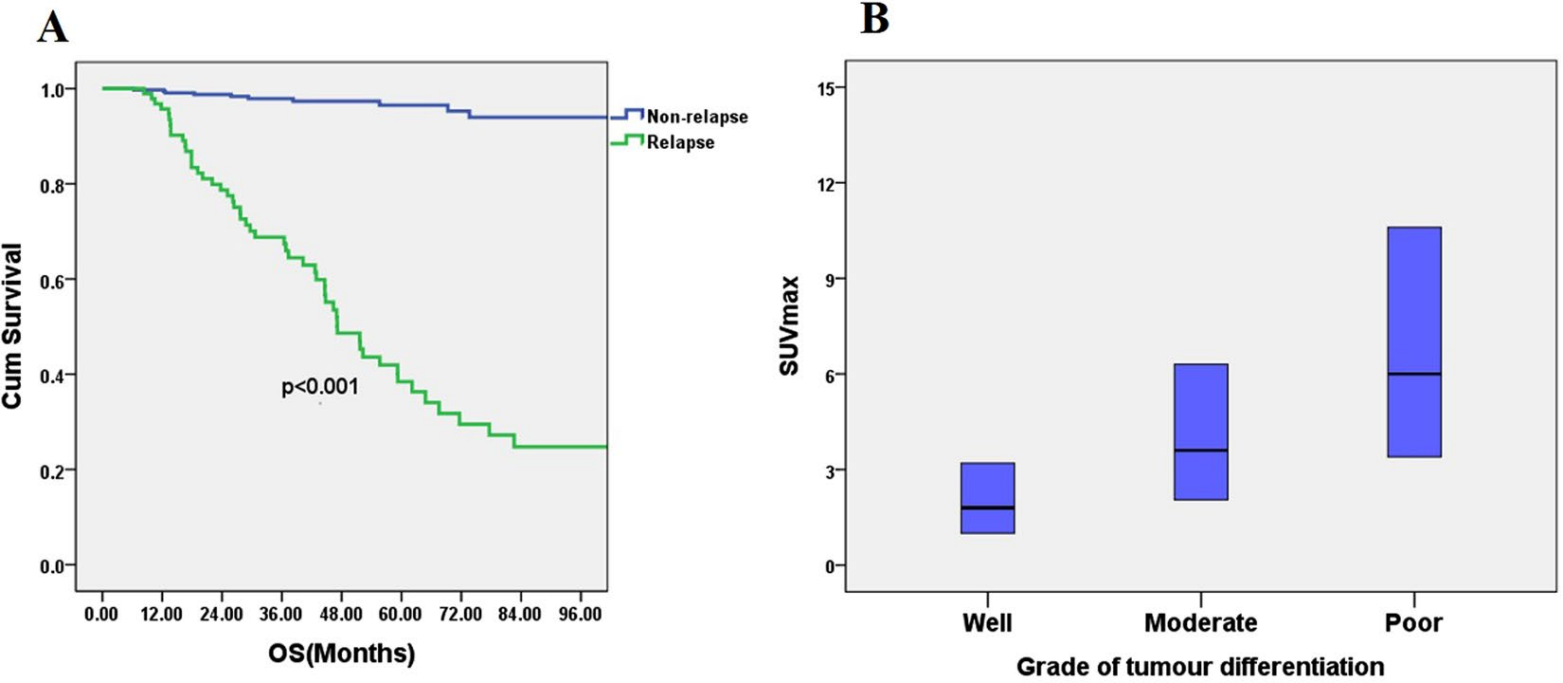
(**A**) Patients relapsed with pulmonary adenocarcinoma also had poor postoperative outcome: five-year overall survival (OS) was 97.3% in the nonrelapse group and 38.4% in the relapse group (*p* < 0.001); (**B**)Tumor differentiation grade correlated with F^18^-fluorodeoxyglucose uptake, expressed as the maximum standard uptake value (SUVmax). The SUVmax of well-differentiated tumors was 2.66 ± 2.64, that of moderately differentiated tumors was 4.75 ± 3.64, and that of poorly differentiated tumors was 7.59 ± 5.25 (*p* < 0.001).

Of 520 lung adenocarcinoma patients, 94 (18.77%) developed relapse after surgery. The common relapses included locoregional recurrence (lung, mediastinum, and pleura) and distant metastasis (brain, bone, liver, and adrenal gland). Sex distribution, age, smoking status, visceral pleural invasion, and serum CEA levels did not differ significantly between the patients with relapse and nonrelapse. In the relapse group, patients had a more advanced pathological stage, more central tumor location. The patients with relapse had more poorly differentiated tumors (*p* < 0.001), a higher percentage of lymphovascular space invasion (22.08%; 7.30% for nonrelapse groups, respectively, *p* < 0.001), higher maximum standard uptake values (SUVmax: 6.09 ± 4.23; 3.74 ± 3.70 for nonrelapse groups, respectively, *p* < 0.001), larger tumor size (2.89 ± 1.35 cm; 2.09 ± 1.29 cm for nonrelapse groups, respectively, *p* < 0.001), and high prevalence of lymph node involvement. The relapse group patients had high prevalence of positive lymph node both for N1 and N2. The results were showed in Tables 1 and 2. Patients developed with relapse had a poor prognosis with a short OS. 5-year OS was 38.4% in the relapse groups, and 97.3% in the nonrelapse groups respectively (*p* < 0.001) (Fig. 1A).

**Table 1:** Characteristics of patients with or without tumor recurrence after resection for clinical stage I or II lung adenocarcinoma.

	No relapse (*n* = 426)	Relapse (*n* = 94)	*p*-value^a^
Sex			
Male	172	44	0.298
Female	254	50	
Differentiation			
Good	224	20	< **0.001**
Moderate	160	45	
Poor	42	29	
Location			
Central	202	58	**0.016**
Peripheral	224	36	
Smoking			
Yes	75	26	0.079
No	321	63	
Ex-smoker	30	5	
Survival			
Yes	414	42	<**0.001**
No	12	52	
LVSI			
Absent	397	77	**0.002**
Present	29	17	
VPI			
Absent	407	91	0.779
Present	19	3	
Operation			
Lobectomy	335	82	**0.031**
Wedge	64	12	
Segmentectomy	27	0	
Status of N1 node			**0.004**
Negative	382	74	
Positive	42	20	
Status of N2 node			<**0.001**
Negative	399	73	
Positive	25	21	
Stage			
0	4	0	<**0.001**
I	265	34	
II	116	29	
III	38	29	
IV	3	2	

^a^Statistically significant *p*-values are shown in bold. LVSI, lymphovascular space invasion; VPI, visceral pleural invasion.

**Table 2:** Characteristics of patients with or without relapse after surgical resection for lung adenocarcinoma.

Variable	No relapse (n = 426)	Relapse (n = 94)	*p*-value^a^
Age (year)	61.60 ± 10.98	62.04 ± 11.53	0.728
SUVmax of tumor	3.74 ± 3.70	6.09 ± 4.23	**<0.001**
Tumor size (cm)	2.09 ± 1.29	2.89 ± 1.35	**<0.001**
CEA (ng/mL)	6.11 ± 27.23	9.13 ± 19.43	0.346
Number of dissected LNs	12.35 ± 7.06	11.47 ± 6.85	0.726

^a^Significance was assessed using Student’s *t* test. SUVmax, maximum standard uptake value; CEA, carcinoembryonic antigen; LNs, lymph nodes.

**Figure 2:**
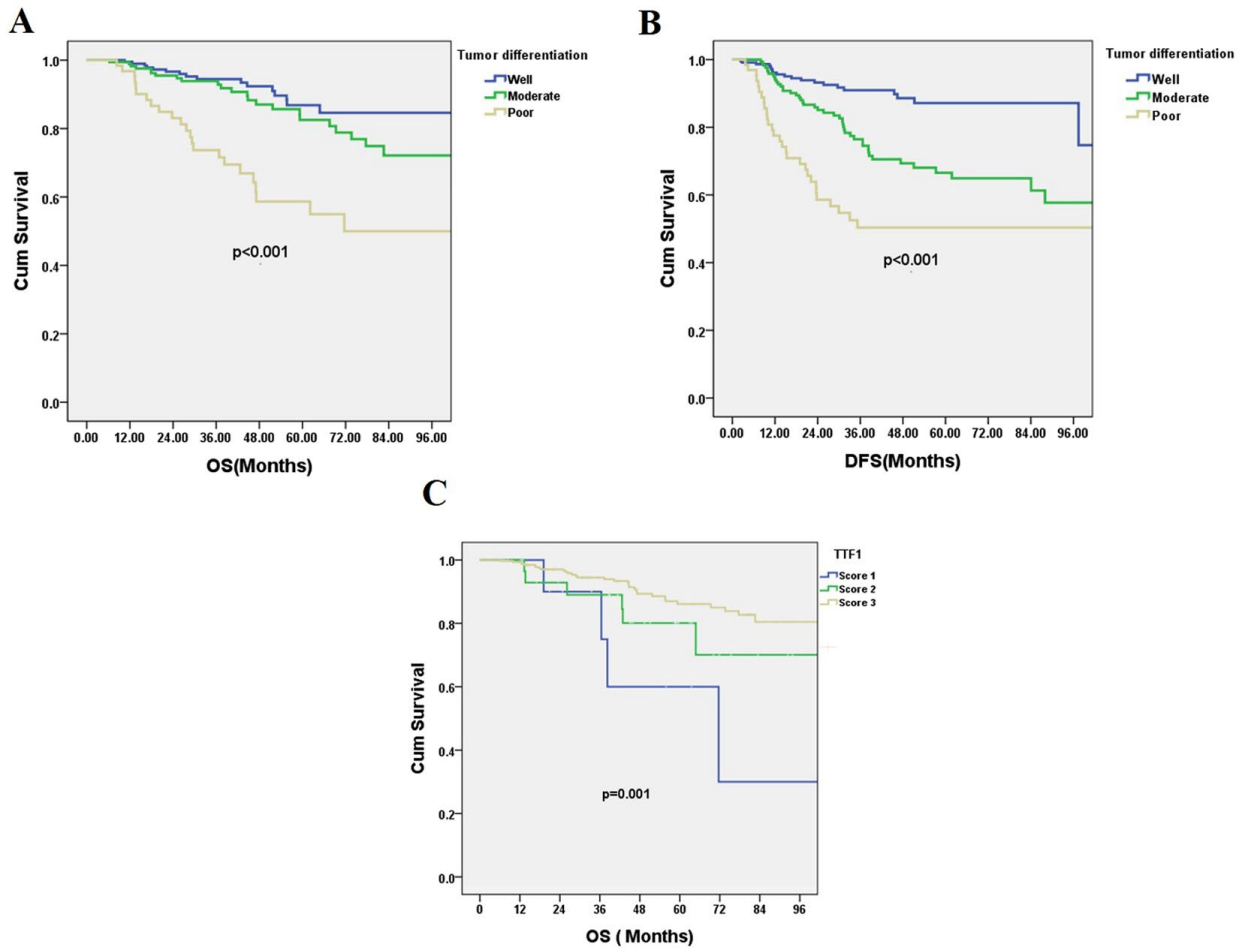
(**A**) Five-year overall survival of patients was 86.8% with well-differentiated tumors, 82.5% with moderately differentiated tumors, and 58.6% with poorly differentiated tumors (*p* < 0.001). (**B**) Five-year disease-free survival of patients was 95.1% with well-differentiated tumors, 66.5% with moderately differentiated tumors, and 50.4% with poorly differentiated tumors, *p* < 0.001; (**C**) The expression of TTF-1 IHC staining was scored after excluded the advanced stage patients (stage 3&4), Five-year OS was 60.0% in score 1 group, 80.1% in score 2 group, and 86.1% in score 3 TTF-1 group.

**Figure 3:**
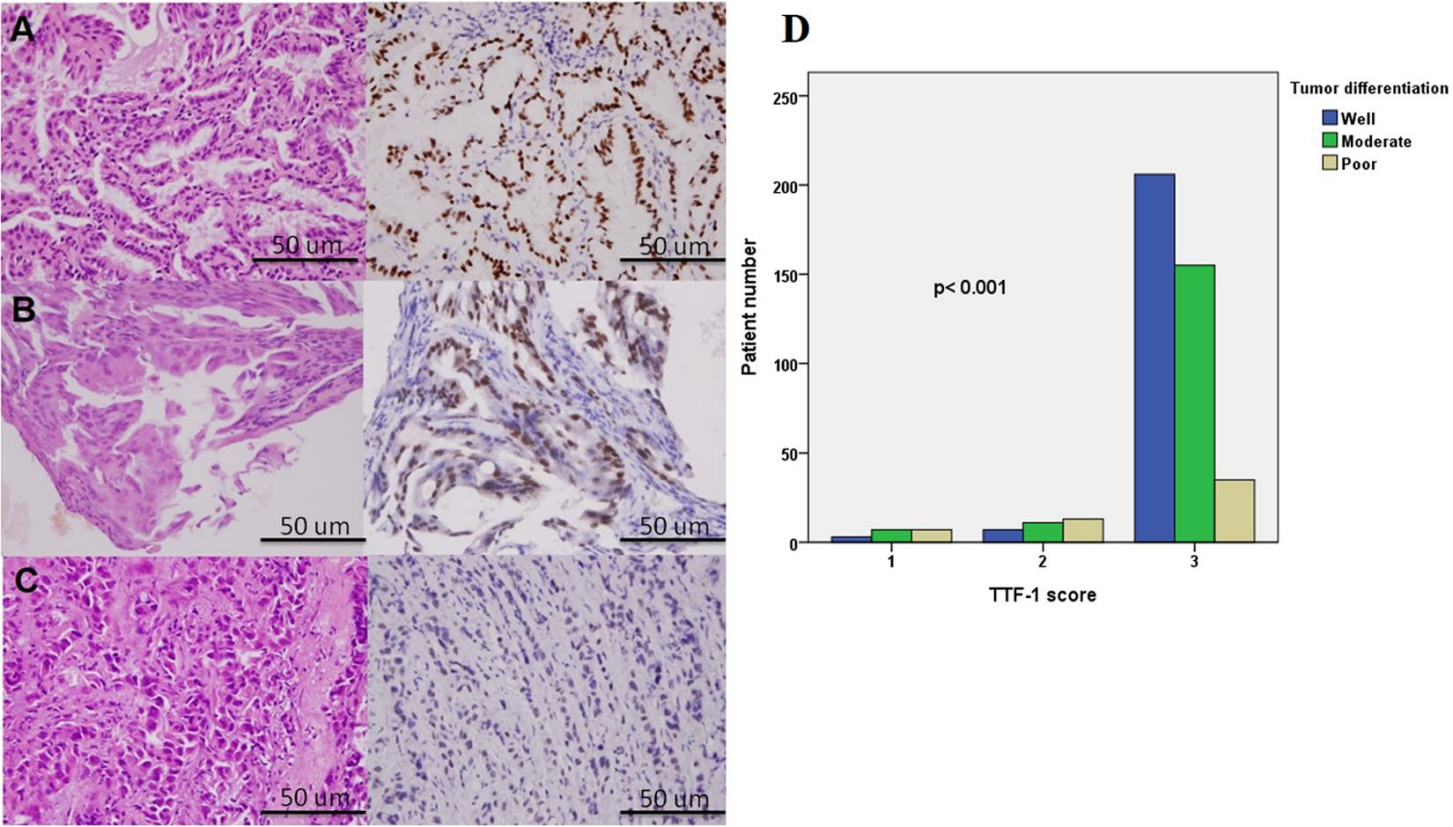
The tumor differentiation grade was also associated with thyroid transcription factor-1 (TTF-1) expression. (**A**) Well-differentiated tumors exhibited stronger TTF-1 expression in immunohistochemical staining (400X). (**B**) Moderately differentiated tumors showed focally positive TTF-1 expression(400X). (**C**) poorly differentiated tumors showed negative TTF-1 expression (400X). (**D**) The χ^2^ test showed TTF-1 score was associated with tumor differentiation (p < 0.001).

**Figure 4:**
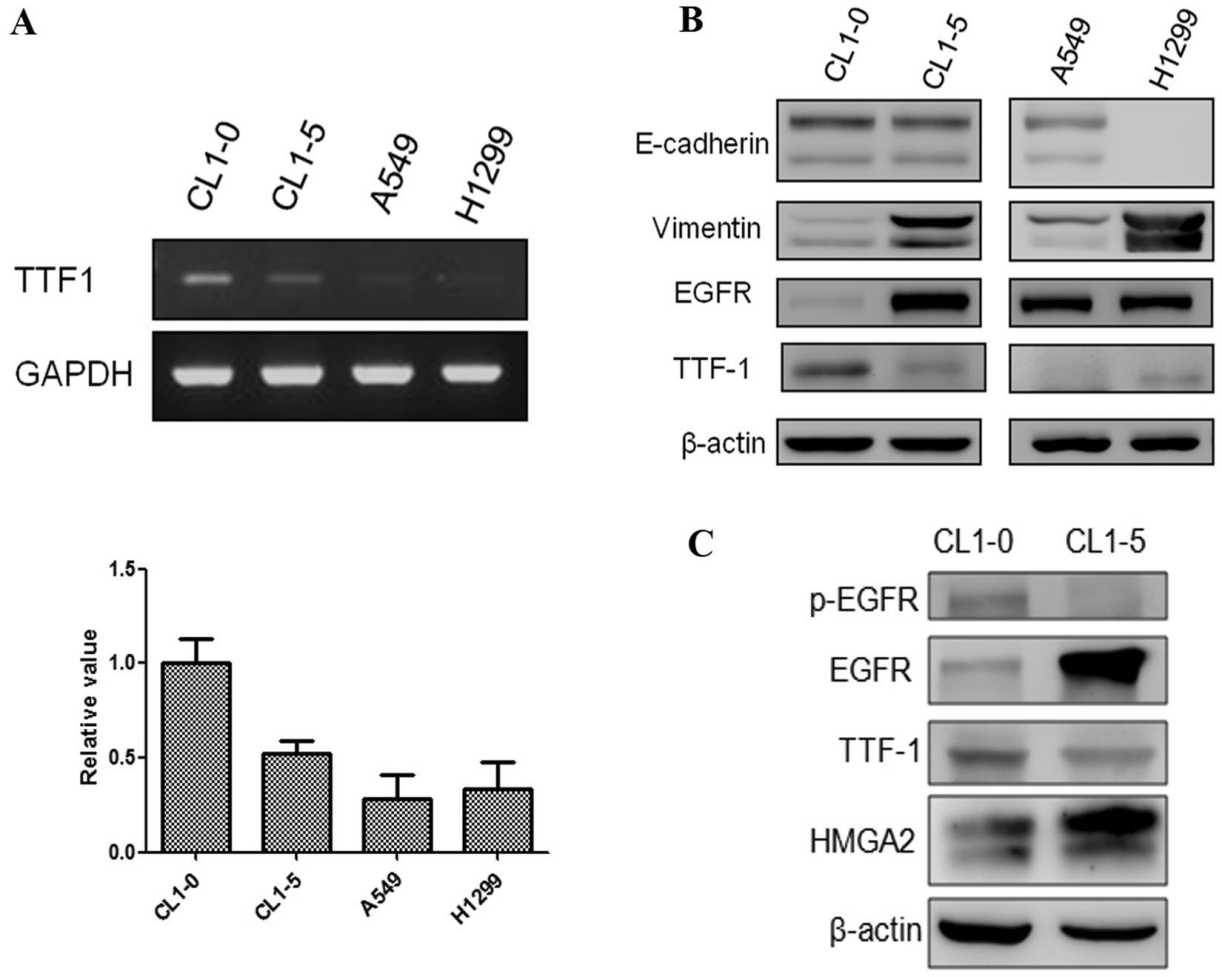
(**A**) Reverse transcription-polymerase chain reaction revealed that TTF-1 expression was significantly higher in CL1-0 cells. (**B**) Western blot analysis validated the difference in tumor aggressiveness between CL1-0, CL1-5, and H1299 cells with different E-cadherin and Vimentin expression. (**C**) The less aggressive cell lines CL1-0 revealed higher TTF-1 expression and lower high-mobility group AT-hook 2 expression. There was reciprocal change in EGFR expression.

Multiple logistic regression revealed that poor tumor differentiation was an independent prognostic factor (odds ratio [OR]: 2.937, 95% confidence interval [CI]: 1.134–7.607; *p* = 0.026). Tumor size was also an independent prognostic factor (OR: 3.371, 95% CI: 1.427–7.959; *p* = 0.006) (Table 3). The grade of tumor differentiation correlated with F^18^-fluorodeoxyglucose uptake, expressed as maximal standard uptake value (SUVmax). The SUVmax of well-differentiated tumors was 2.66 ± 2.64, that of moderately differentiated tumors was 4.75 ± 3.64, and that of poorly differentiated tumors was 7.59 ± 5.25 (*p* < 0.001) (Fig. 1B). OS and DFS significantly correlated with the grade of tumor differentiation. 5-year OS was 86.8% in the well-differentiated group, 82.5% in the moderately differentiated group, and 58.6% in the poorly differentiated group, *p* < 0.001 (Fig. 2A). 5-year DFS was 95.1% in the well-differentiated group, 66.5% in the moderately differentiated group, and 50.4% in the poorly differentiated group, *p* < 0.001 (Fig. 2B). The expression of TTF-1 IHC staining was scored after excluded advanced after excluding advanced disease (pathological stage 3 & 4). 5-year OS was 60.0% in score 1 group, 80.1% in score 2 group, and 86.1% in score 3 TTF-1 group (Fig. 2C). Well-differentiated tumors exhibited stronger TTF-1 expression in IHC stain (Fig. 3A). Moderately differentiated tumors showed focally positive TTF-1 expression (Fig. 3B). Poorly differentiated tumors showed negative TTF-1 expression (Fig. 3C). The expression of TTF-1 was associated with tumor differentiation in resected lung adenocarcinoma patients (*p* < 0.001, Fig. 3D). The result was validated via lung cancer cell line study. CL1-0 cells exhibited stronger expression of TTF-1 at both the RNA and protein levels compared with CL1-5 cells (Fig. 4A). CL1-5 cells exhibited more aggressive behavior. Levels of E-cadherin, vimentin as shown in Western blot analysis confirmed the tumor aggressiveness of CL1-5 cells (Fig. 4B). The less aggressive cell lines CL1-0 had higher TTF-1 expression and lower high-mobility group AT-hook 2 expression. It revealed reciprocal change in epidermal growth factor receptor (EGFR) expression (Fig. 4C).

**Table 3:** Multiple logistic regression analysis of postoperative relapse for patients with pathological stage I & II lung adenocarcinoma.

Factor	OR (95% CI)	p-value
SUVmax > 4	1.625 (0.777–3.399)	0.197
Tumor size >2 cm	3.371 (1.427–7.959)	**0.006**
Differentiation		
Well	Reference	
Moderate	1.324 (0.579–3.028)	0.505
Poor	2.937 (1.134–7.607)	**0.026**
LVSI	1.126 (0.708–1.793)	0.616
Operation		
Sublobar resection	0.855 (0.285–2.835)	0.797
Status of N1 node		
Positive	1.682 (0.741–3.820)	0.214

NSCLC, non-small-cell lung cancer; OR, odds ratio; CI, confidence interval; SUVmax, maximum standard uptake value; LVSI, lymphovascular space invasion.

**Figure 5:**
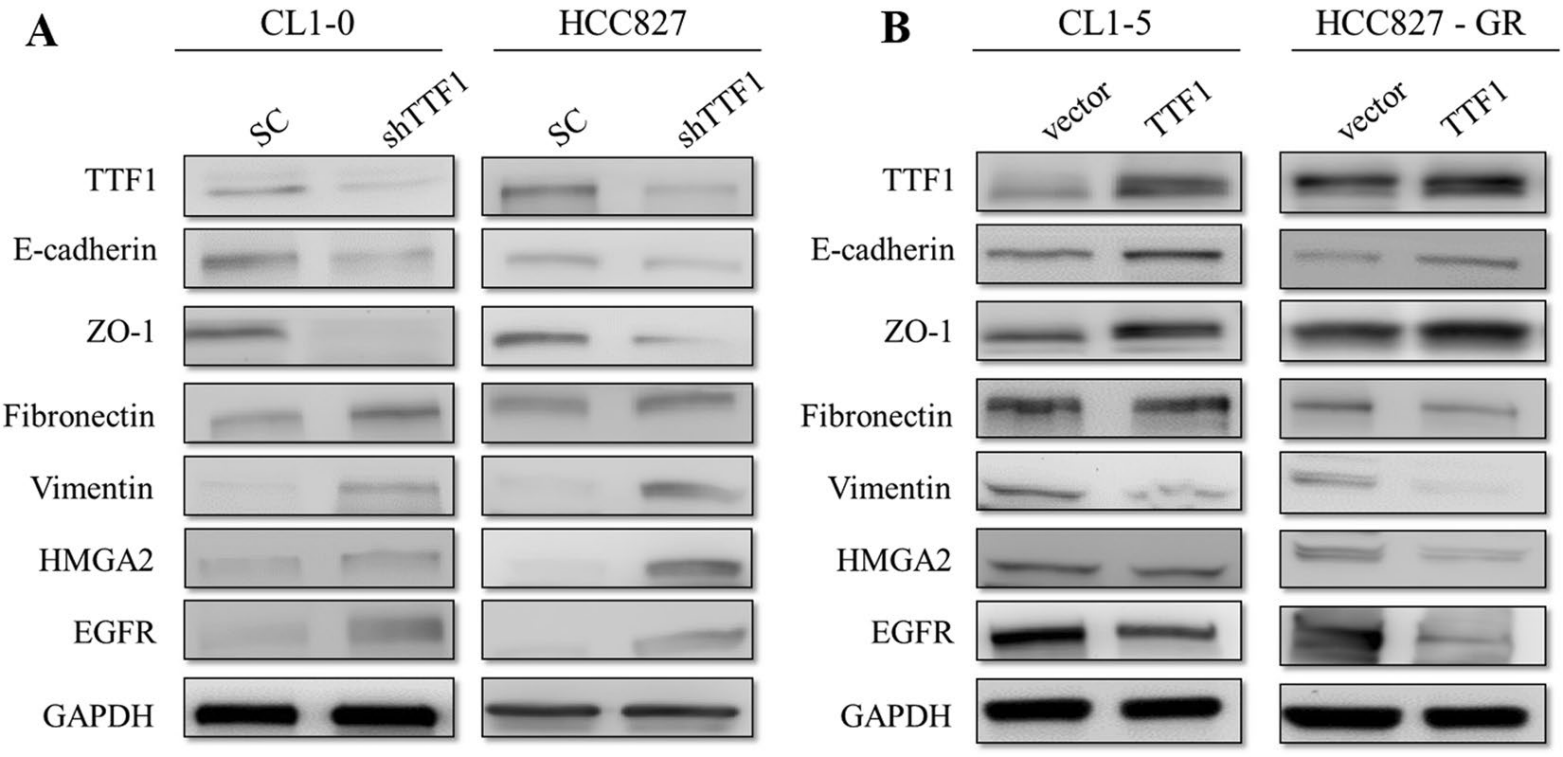
The expression of TTF-1, HMGA2, EMT markers and EGFR in CL1-0-SC, CL1-0-shTTF1, HCC827-SC, HCC827-shTTF1, CL1-5-Vector, CL1-5-TTF1, HCC827-GR-Vector and HCC827-GR-TTF1 cells were evaluated by western blotting. (**A**) The expression of HMGA2, mesenchymal markers and EGFR increased in CL1-0 and HCC827 cells but expression of epithelial markers decreased after knocked down TTF-1 by shRNA. (**B**) The expression of HMGA2, mesenchymal markers and EGFR decreased but expression of epithelial markers increased in CL1-5 and HCC827-GR cells following overexpression of TTF-1 by human TTF-1 cDNA ORF clone. The Western blotting was independently repeated three times, and the representative data are shown. GAPDH was served as a loading control.

**Figure 6:**
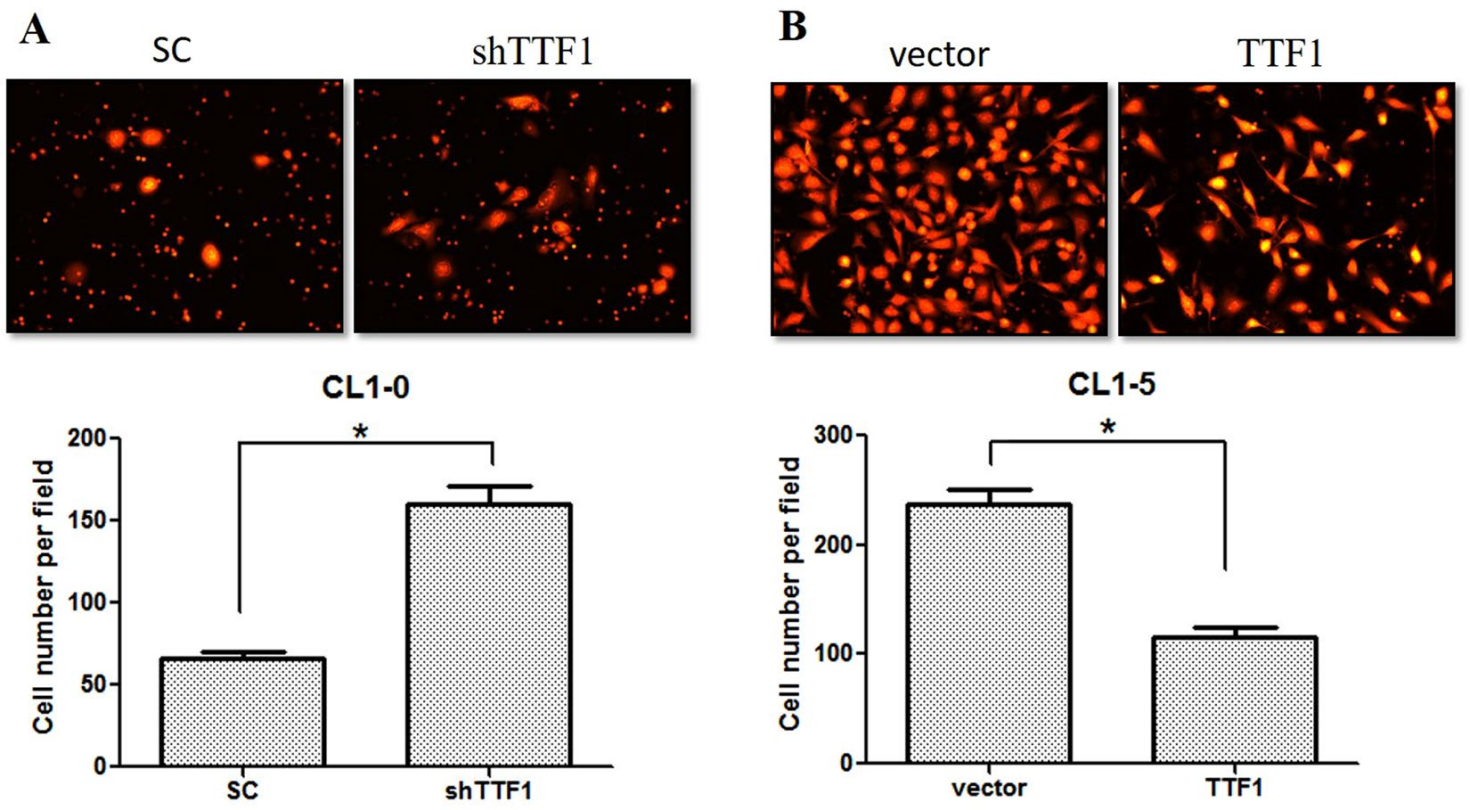
The invasion abilities of CL1-0 and CL1-5 cells following knockdown/overexpression of TTF-1 were determined by transwell invasion assays. The invasion abilities were significantly inversely correlated with the expression of TTF-1. The transwell invasion assay was independently performed three times, and the representative data are shown. *Statistically significant differences between Scramble control/Vector and TTF-1 knockdown/overexpression group, p < 0.05 by Student’s t test. Error bars, S.E.M. of three independent experiments.

**Figure 7:**
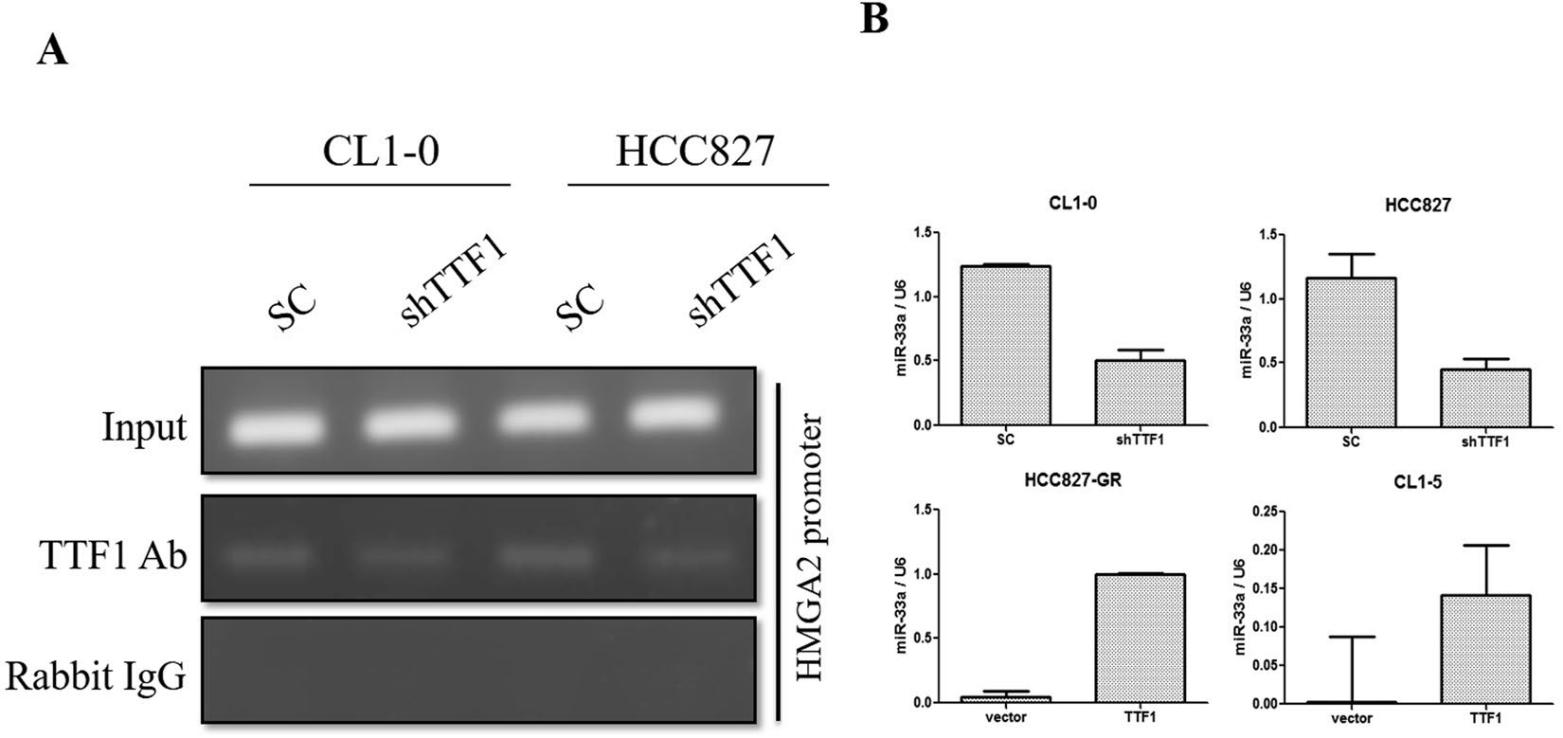
(**A**) Total lysates from CL1-0-SC, CL1-0-shTTF1, HCC827-SC and HCC827-shTTF1 cells were immunoprecipitated using 5 μg of anti-TTF-1 antibody or normal rabbit IgG and then incubated with magnetic beads. The precipitated chromatin was analyzed by PCR to investigate the interaction between TTF-1 and HMGA2 promoter. TTF-1 co-immunoprecipitated with HMGA2 promoter containing TTF1-recognition site and the effect was blocked after knockdown TTF-1 by TTF-1 shRNA. The results shown are representative of three independent experiments. (**B**) The miR33a expression was quantified by qPCR in CL1-0-SC, CL1-0-shTTF1, HCC827-SC, HCC827-shTTF1, CL1-5-Vector, CL1-5-TTF1, HCC827-GR-Vector and HCC827-GR-TTF1 cells. The expression of miR-33a was positively correlated with TTF-1 expression and this was validated by knockdown/overexpression of TTF-1. U6 was served as a loading control. Error bars, S.E.M. of three independent experiments.

The expression of HMGA2, mesenchymal markers and EGFR increased in CL1-0 and HCC827 cells but expression of epithelial markers decreased after knocked down TTF-1 by shRNA (Fig. 5A). The expression of HMGA2, mesenchymal markers and EGFR decreased but expression of epithelial markers increased in CL1-5 and HCC827-GR cells following overexpression of TTF-1 by human TTF-1 cDNA ORF clone (Fig. 5B). The invasion ability of CL1-0 cells increased when knockdown TTF-1 expression with shTTF-1. In contrast, the invasion ability of CL1-5 cells decreased when overexpression of TTF-1(Fig. 6A and B). The invasion abilities were significantly inversely correlated with the expression of TTF-1. The precipitated chromatin was analyzed by PCR to investigate the interaction between TTF-1 and HMGA2 promoter. TTF-1 co-immunoprecipitated with HMGA2 promoter containing TTF1-recognition site and the effect was blocked after knockdown TTF-1 by TTF-1 shRNA (Fig. 7A). The expression of miR-33a was positively correlated with TTF-1 expression and this was validated by knockdown and overexpression of TTF-1 (Fig. 7B).

## Discussion

Lung cancer is the leading cause of cancer deaths worldwide. Recurrent rate after complete resection for patients with stage I disease was ranged from 25% to 50%^18^. Some studies have reported that poor tumor differentiation is an independent prognostic factor in patients with stage I NSCLC^3,4^. However, other studies have shown that differentiation grade is not a prognostic factor for lung cancer survival^12,13^. The role of tumor histological grade as a prognostic factor is controversial.

The molecular mechanisms underlying tumor cell differentiation and the biological effects of prognosis are not clear. In previous study, poor differentiation of tumors was an independent prognostic factor for patients with pathological stage I NSCLC after surgical resection^19^. The grade of tumor differentiation as a prognostic factor in pulmonary adenocarcinoma has not been well clarified. In this study, we found that tumor differentiation was a prognostic factor for adenocarcinoma patients who underwent surgical resection. Poor tumor differentiation was an independent prognostic factor with (OR = 2.937). The result of metabolic PET scan supported this finding. Poorly differentiated tumors had a higher SUVmax of glucose uptake than moderately and well-differentiated tumors. This result is compatible with the metabolic concept of tumor biology.

TTF-1 plays key roles in the control of tissue-specific gene expression and in lung morphogenesis and differentiation^5^. Haploinsufficiency of TTF-1 is associated with tumor formation in transgenic mice^20^. TTF-1 expression is higher in lung adenocarcinoma than in normal lung tissue but decrease in metastatic lesions^15^. TTF-1 is enigmatic with dual roles in cancer cell survival and progression^14^. The mechanisms by which TTF-1 and its transcriptional activity are related to tumor aggressiveness are not fully understood. In clinical observations, the TTF-1 expression presented reciprocal correlation with the grade of tumor differentiation (Fig. 3). After scoring the TTF-1 expression for 520 resected lung adenocarcinoma patients, the expression of TTF-1 was correlated with tumor differentiation (*p* < 0.001). In this study, tumor differentiation was associated with TTF-1 expression and resulted in different surgical outcomes. The conclusion based on the clinical data was confirmed through *in vitro* studies of lung cancer cell lines. The expression of TTF-1 at both the RNA and protein level were high in CL1-0 cells and low in CL1-5 cells. CL1-5 cells displayed more aggressive behavior than CL1-0 cells. Western blot analysis of E-cadherin, vimentin, and HMGA2 expression confirmed the tumor aggressiveness of CL1-5 cells. The less aggressive cell lines (CL1-0) had higher TTF-1 expression and lower high-mobility group AT-hook 2 expression.

The highly invasive tumor cell line (CL1-5) had strong HMGA2 expression. HMGA2 is a member of the high-mobility group protein family. It plays a critical role in the invasion and metastasis of human cancers^21^ and is involved in the epithelial–mesenchymal transition (EMT) process^22^. The functional studies via knockdown and overexpression of TTF-1 resulted in the respondent change of EMT marker, HMGA2 expression and the invasion ability. How does TTF-1 expression involve in the regulation of HMGA2 expression? Suppression of lung adenocarcinoma progression by TTF-1 via regulation of HMGA2 has been reported^15^. However, the mechanism by which TTF-1 regulates HGMA2 is not well known. In this study, TTF-1 co-immunoprecipitated with HMGA2 promoter containing TTF1-recognition site and the effect was blocked after knockdown TTF-1 by TTF-1 shRNA. The possible mechanism of TTF-1 involved in the regulation of HMGA2 expression via direct binding to promotor region. In addition, the expression of miR-33a was positively correlated with TTF-1 expression and this was validated by knockdown/overexpression of TTF-1. Micro RNAs are small non-protein-coding RNA that regulate gene expression by binding to the target mRNA. The miR 365 regulates lung cancer and development gene TTF-1 was reported^23^. The miR33a expression decreased after the suppressive effect of TTF-1. Our data suggest that TTF-1 is an important regulator of miR 33a. The TTF-1, miR33a and HMGA2 may have cross talk in regulation of the lung adenocarcinoma. The further study to investigate the miR33a will be worked.

In this study, TTF-1 expression was also associated with EGFR expression in lung adenocarcinoma cell lines. TTF-1-induced receptor tyrosine kinase-like orphan receptor 1 is required to sustain EGFR survival signaling in lung adenocarcinoma^24^. Transforming growth factor β1 (TGF-β1) induces stemness and plasticity through the EGFR/Ras signaling pathway and specifically activates HMGA2^25^. The TGF-β1 induction of HMGA2 is abrogated by EGFR inhibitors^26^. A blockade of TGF signaling induces a subset of genes that regulate hormone-induced type II cell differentiation, including TTF-1^27^. Tumor-associated macrophages also promote the EMT process in tumor cells by producing TGF-β. An analysis of 491 patients with NSCLC revealed positive correlations between intratumoral macrophage density, EMT markers, intraepithelial TGF-β levels, and tumor grade^28^. We speculated that tumor-associated macrophages may regulate TTF-1 expression by producing TGF-β and that the TTF-1/HMGA2/EFGR pathway may play a role in the carcinogenesis of lung adenocarcinoma. Further work is needed to clarify this hypothesis.

The limitations of this study are that it was a single-institution retrospective study. The study focused on the lung adenocarcinoma. The subtype of lung adenocarcinoma did not dissect clearly. The mucinous type adenocarcinoma had different TTF-1 expression. Further study should be done to clarify the different mechanism of different subtype adenocarcinoma in this issue. In addition, the protein and RNA expression of clinical patient’s specimen did not demonstrated. Further study should be conducted in clarify this issue.

## Conclusion

The grade of tumor differentiation is a crucial prognostic factor in lung adenocarcinoma. TTF-1/HMGA2 asix is associated with tumor differentiation and aggressiveness. TTF-1-mediated tumor differentiation is involved in the tumorigenesis of pulmonary adenocarcinoma.
